# Long-term expression of miRNA for RNA interference using a novel vector system based on a negative-strand RNA virus

**DOI:** 10.1038/srep26154

**Published:** 2016-05-18

**Authors:** Tomoyuki Honda, Yusuke Yamamoto, Takuji Daito, Yusuke Matsumoto, Akiko Makino, Keizo Tomonaga

**Affiliations:** 1 Department of Viral Oncology, Institute for Virus Research, Kyoto University, Kyoto 606-8507, Japan; 2 Department of Mammalian Regulatory Network, Graduate School of Biostudies, Kyoto University, Kyoto 606-8507, Japan; 3 Center for Emerging Virus Research, Institute for Virus Research, Kyoto University, Kyoto 606-8507, Japan; 4 Department of Tumor Viruses, Graduate School of Medicine, Kyoto University, Kyoto, Kyoto 606-8507, Japan

## Abstract

RNA interference (RNAi) has emerged as a promising technique for gene therapy. However, the safe and long-term expression of small RNA molecules is a major concern for the application of RNAi therapies *in vivo*. Borna disease virus (BDV), a non-segmented, negative-strand RNA virus, establishes a persistent infection without obvious cytopathic effects. Unique among animal non-retroviral RNA viruses, BDV persistently establishes a long-lasting persistent infection in the nucleus. These features make BDV ideal for RNA virus vector persistently expressing small RNAs. Here, we demonstrated that the recombinant BDV (rBDV) containing the miR-155 precursor, rBDV-miR-155, persistently expressed miR-155 and efficiently silenced its target gene. The stem region of the miR-155 precursor in rBDV-miR-155 was replaceable by any miRNA sequences of interest and that such rBDVs efficiently silence the expression of target genes. Collectively, BDV vector would be a novel RNA virus vector enabling the long-term expression of miRNAs for RNAi therapies.

Small RNA molecules, such as small interfering RNAs (siRNA), microRNAs (miRNA) and short-hairpin RNAs (shRNA), are central to RNA interference (RNAi), a naturally occurring regulatory mechanism causing posttranscriptional gene silencing in most eukaryotic cells^1^,^2^. siRNAs are short (21- to 25-nucleotides [nt]) dsRNAs that mediate mRNA degradation in a sequence-specific manner^3^. Long-dsRNAs are cleaved by the cellular RNase III family member, Dicer, into short dsRNA duplexes in the cytoplasm^4^. miRNAs are non-coding, single-stranded RNAs transcribed by RNA polymerase II from sequences encoded in the genome^1^,^4^. The biogenesis of miRNAs begins with the transcription of a long primary miRNA (pri-miRNA) that is processed in the nucleus into a 70- to 80-nt hairpin miRNA (pre-miRNA) by the cellular RNase III enzyme, Drosha, and its co-factor, DiGeorge syndrome critical region gene 8 (DGCR8)^1^,^4^. The pre-miRNA is further processed by Dicer, which cleaves the bulge of the pre-miRNA to produce a mature miRNA of ~22-nt^1^,^4^. shRNAs are RNA sequences with a tight hairpin loop and are transcribed from shRNA-expressing constructs and processed into siRNAs by the Dicer-mediated cleavage of the hairpin structure in the cytoplasm. All three types of small RNAs enter the RNA-induced silencing complex (RISC) in the cytoplasm. siRNAs and shRNAs degrade target mRNAs with perfect complementary matching sequences, whereas miRNAs mostly need complementarity only in the seed sequence to target RNAs for translational repression and/or degradation^5^.

Posttranscriptional gene regulation by RNAi is a promising approach to gene-specific suppression and new therapeutic strategies against various diseases. At present, however, RNAi-based therapies have largely not been practical, because of a lack of tools for delivering these small RNA molecules efficiently into the target cells. Thus, one of the major challenges of RNAi-based therapies is the delivery of the RNAi molecules to the correct cells at the right time. Furthermore, stable and secure expression of these micromolecules in the cells is required for the RNAi therapies to be effective *in vivo*^6^. Although siRNAs can be introduced into cells as synthetic oligonucleotides, vector-derived expression of miRNAs would be more useful for the efficient and persistent expression of small RNA molecules because a vector-derived transcript can be processed into multiple, functional miRNAs.

Currently, viral vector systems are used widely for the expression of miRNAs and shRNAs. Adeno-associated virus (AAV), adenovirus and retrovirus vectors are commonly used as delivery systems for these small RNA molecules. Although these systems may express the micromolecules effectively, each vector has specific limitations that restrict its application. For example, it is known that AAV and adenovirus vectors are gradually diluted out in dividing cells and, therefore, are not suitable for the long-term expression of small RNAs^7^. Adenovirus vectors are known to induce cytopathic effects (CPEs) and strong cellular immune responses^7^. Lentivirus vectors may cause oncogenic mutations by their integration into the host genome^6^,^7^. Furthermore, transgene expression from the integrated vectors may be transient, due to the host epigenetic silencing^7^,^8^,^9^. Therefore, the development of a new viral vector system that enables efficient, stable and secure expression of RNAi molecules is required for the further development of RNAi technologies.

Vectors based on negative-strand RNA viruses (NSV) for expression of small RNAs have been reported rarely because almost all RNA viruses are transcribed in the cytoplasm, whereas the processing machinery of pri-miRNAs is in the nucleus. Recently, we established a novel RNA virus-based vector using Borna disease virus (BDV) reverse genetics^10^,^11^. This BDV vector harbors an extra transcription cassette in the intercistronic non-coding region between the viral phosphoprotein (P) and matrix (M) genes and can express foreign genes efficiently and stably in infected cells. BDV is a non-segmented NSV and it readily establishes a long-lasting, persistent infection in various types of cells without overt CPEs^12^,^13^. A striking feature of BDV is that it replicates in the nuclei of infected cells^12^,^13^,^14^,^15^. These characteristics make BDV the only animal non-retroviral RNA virus capable of intranuclear parasitism, offering the possibility that it may be an ideal candidate for the long-term expression system of small RNA.

Here, we report the establishment of a novel BDV vector carrying a pri-miRNA-cassette sequence in an intercistronic, noncoding region of the viral genome. The recombinant BDV (rBDV), rBDV-miR-155, containing the pri-miR-155 sequence, stably expressed miR-155 in cultured cells for a long period of time and efficiently silenced a reporter gene containing the miR-155 target sequence in the 3′-untranslated region (UTR). Furthermore, downregulation of Dicer abrogated silencing of the target by rBDV-miR-155. We also demonstrate that the pre-miRNA sequence in the pri-miR-155-cassette is replaceable by any miRNA sequences of interest and that such BDV vectors, named miBDV vectors, can efficiently suppress the expression of endogenous and exogenous target genes. Our results show that the BDV vector can be used for the delivery and long-term expression of small RNA molecules and should provide a novel tool for RNAi therapies.

## Results

### Generation of a BDV vector expressing pri-miRNA from an intercistronic region of the genome

To express small RNAs from the BDV vector, we constructed pBDV plasmids harboring a pri-miRNA sequence in an intercistronic region between the P and M genes of the BDV genome. To test miRNA expression, we chose the mouse miR-155, because the sequence information for this miRNA and the expression profiles have been well documented in previous studies^16^,^17^,^18^. The minimum 150-nt sequence required for miR-155 expression was subcloned into the region between the P and M genes in the pBDV plasmid using the *Bst* BI and *Pac* I restriction sites of the cassette region (Supplemental Fig. 1A).

**Figure 1:**
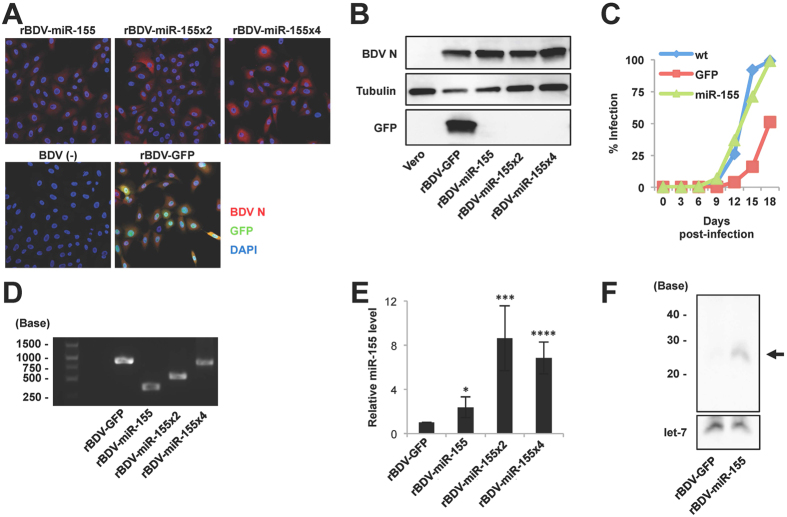
Generation of rBDVs expressing miRNA. (**A,B**) Replication of rBDVs carrying the pri-miR-155 sequences. BDV infection was evaluated by IFA (**A**) and western blot analysis (**B**) using the indicated antibodies. (**C**) Growth kinetics of wt, rBDV-GFP and rBDV-miR-155. Vero cells were infected with rBDVs at an MOI of 0.01 and the viral growth rate was monitored by IFA. (**D**) Stability of the pri-miR-155 cassette in rBDV-miR-155. Copies of pri-miR-155 were detected by PCR analysis in Vero cells at 6 months post-infection. (**E**) Quantification of miR-155 expression in rBDV-miR-155-infected Vero cells. The expression of miR-155 was measured and standardized by that of β-actin. (**F**) Northern blot analysis of rBDV-derived miR-155. The expression of let-7 was used as a loading control. Full length blots of (**B**,**D**,**F**) are presented in Supplemental Fig. 4. Values are expressed as the mean + S.E. *P < 0.05; ***P < 0.005; ****P < 0.001.

We tried to produce the rBDV carrying the pri-miR-155 sequence (rBDV-miR-155) using a standard BDV reverse genetics protocol established previously (see Materials and methods)^10^. A few weeks after co-cultivation with Vero cells, we obtained rBDV-miRNA-155 (Fig. 1A,B). As shown in Fig. 1C, the growth kinetics of rBDV-miR-155 and wild-type (wt) rBDV were comparable. On the other hand, consistent with our previous study^10^, the growth of rBDV expressing GFP (rBDV-GFP) was slightly slower than wt rBDV (Fig. 1C). We also constructed pBDVs carrying two, four and eight tandem copies of the pri-miR-155 sequences, pBDV-miR-155x2, pBDV-miR-155x4 and pBDV-miR-155x8, respectively (Supplemental Fig. 1A), and successfully obtained rBDVs carrying two and four, but not eight, tandem copies of the pri-miR-155 sequence (Fig. 1A,B). The tandem pri-miR-155 sequences were retained in the genome for at least 6 months in the cells infected persistently with rBDVs (Fig. 1D). Robust expression of miR-155 in rBDV-miR-155-infected Vero cells was detected by mouse miR-155-specific qRT-PCR analysis (Fig. 1E). Furthermore, northern blot analysis confirmed substantial expression of mature miR-155 in rBDV-miR-155-infected cells (Fig. 1F).

### Expression of functional miRNA using BDV vectors

**Figure 2:**
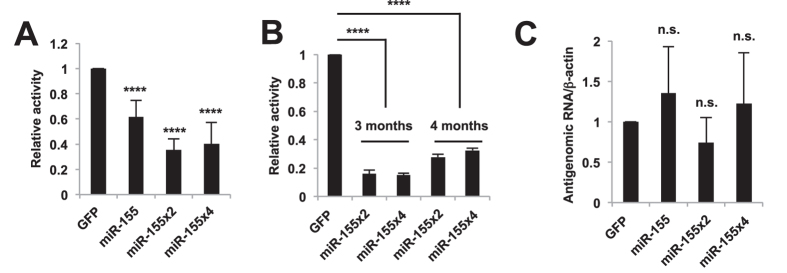
Expression of functional miR-155 in rBDV-miR-155-infected cells. (**A**) Relative luciferase activity after transfection of pLuc-miR-155as into rBDV-miR-155-infected Vero cells. (**B**) Relative luciferase activity at 3 or 4 months post-infection with rBDV-miR-155x2 and x4. (**C**) Quantification of BDV antigenomic RNA expression in rBDV-miR-155-infected Vero cells. The level of BDV antigenomic RNA was measured and standardized with that of β-actin. Values are expressed as the mean + S.E. ****P < 0.001; n.s., no significance.

To determine whether functional miRNAs are expressed from BDV vectors, we generated a luciferase reporter plasmid, pLuc-miR-155as, containing an artificial 22-nt sequence complementary to miR-155 inserted into the 3′-UTR of the luciferase gene (Supplemental Fig. 1B), and transfected this reporter plasmid into the rBDV-miR-155-infected cells. As shown in Fig. 2A, the luciferase activity was reduced in the cells infected with rBDV expressing miRNAs. Notably, gene silencing of rBDV-miR-155x2 and rBDV-miR-155x4 seemed to be efficient, compared to rBDV-miR-155 (Fig. 2A), consistent with our data shown in Fig. 1E. The silencing efficacy by rBDV-miR-155x2 and rBDV-miR-155x4 were comparable to that by transfection of mouse miR-155 mimic or infection of the lentiviral vector expressing miR-155, whereas rBDV-miR-155 silenced the luciferase reporter less efficiently (Fig. 2A,B and Supplemental Fig. 1C,D). To ensure that virus-derived miR-155 was produced by the endogenous miRNA machinery, we examined the gene silencing of rBDV-miR-155 in the presence of siRNA against Dicer (siDicer). The silencing effect of rBDV-miR-155 was abrogated by Dicer knockdown (Supplemental Fig. 2A). Silencing of the target gene by rBDV-miR-155x2 and rBDV-miR-155x4 was still detectable at 3 or 4 months post-infection (Fig. 2B). These results indicate that the BDV vector can produce a functional mature miRNA for a long period of time in the infected cells through the canonical miRNA processing pathway and that multimerization of pri-miRNA sequences in the BDV vector may increase the silencing of the target mRNA as efficient as that by current methods.

To determine whether the functional miRNA in the rBDV-miR-155-infected cells was expressed from antigenomic RNA or mRNA encoding the pri-miR-155 sequence, we measured the levels of BDV RNAs. If the mature miRNAs were produced directly from the pri-mRNA sequences in the antigenomic RNA by Drosha, the level of BDV antigenome RNA should be reduced in the rBDV-miR-155-infected cells. As shown in Fig. 2C and Supplemental Fig. 2B, the level of BDV antigenomic RNA was comparable between rBDV-GFP- and rBDV-miR-155-infected cells, suggesting that BDV antigenomic RNA may not be a substrate for Drosha-mediated cleavage and, therefore, that the mRNA encoding the pri-miR-155 sequences could be the substrate. This idea is consistent with the previous studies showing the ability to express functional miR-155 from heterologous RNA polymerase II promoter^16^,^18^.

### Generation of miBDV vector expressing miRNAs of interest

**Figure 3:**
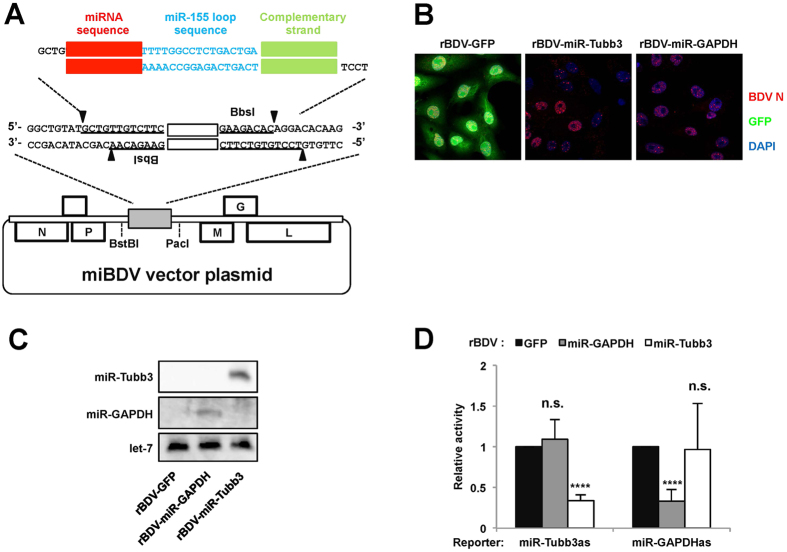
Development of a miBDV vector system. (**A**) Schematic representation of a miBDV vector containing a cassette site for the insertion of miRNAs of interest. (**B**) Evaluation of BDV infection by IFA. (**C**) Northern blot analysis of rBDV-derived miRNAs. The expression of let-7 was used as a loading control. (**D**) Relative luciferase activity after transfection of pLuc-miR-Tubb3as or pLuc-miR-GAPDHas into Vero cells infected with rBDV-miR-Tubb3 or rBDV-miR-GAPDH. Full length blots of (**C**) are presented in Supplemental Fig. 4. Values are expressed as the mean + S.E. ****P < 0.001; n.s., no significance.

We sought next to generate miBDV vectors, which can express miRNAs of interest from the intercistronic region, by modification of pBDV-miR-155. It has been reported that stem sequences of pre-miRNAs do not contribute to mature miRNA production and that it is possible to produce novel artificial miRNAs by substituting the stem sequences of pre-miRNAs^19^. Based on this idea, we introduced *Bbs* I sites into the pri-miR-155 region of pBDV-miR-155, which enable the replacement of pre-miR-155 with the pre-miRNA of any miRNA sequences of interest (Fig. 3A). The miRNA of interest can be inserted into the *Bbs* I sites of this pBDV vector plasmid as a synthetic 64-nt DNA duplex, which encodes a pre-miRNA stem sequence within the miR-155 loop region (Fig. 3A and Supplemental Fig. 3). As models of artificial miRNAs, we introduced pre-miRNA stem sequences designed to target the mouse tubulin β3 and GAPDH mRNAs into the cassette region and generated pBDV-miR-Tubb3 and pBDV-miR-GAPDH, respectively (Supplemental Fig. 3).

Using these pBDVs, we rescued rBDVs, rBDV-miR-Tubb3 and rBDV-miR-GAPDH, which were expected to produce artificial 22-nt miRNAs complementary to the target mRNAs (Fig. 3B). We confirmed that these rBDVs indeed produced artificial miRNAs that we designed by northern blot and the designed miRNA-specific qRT-PCR analyses (Fig. 3C and Supplemental Fig. 3B). To determine whether these miBDV vectors produce functional artificial miRNAs against their targets, we transfected the reporter plasmids, pLuc-miR-Tubb3as and pLuc-miR-GAPDHas, which contain the target sequence of the miRNAs in the 3′-UTR of the luciferase gene, into rBDV-infected Vero cells. As shown in Fig. 3D, the miRNA-expressing rBDVs significantly reduced the luciferase activity of the corresponding reporter plasmid, indicating that the artificial miRNAs of interest can be expressed from miBDV vectors using the pri-miR-155 sequence as a backbone of the cassette.

**Figure 4:**
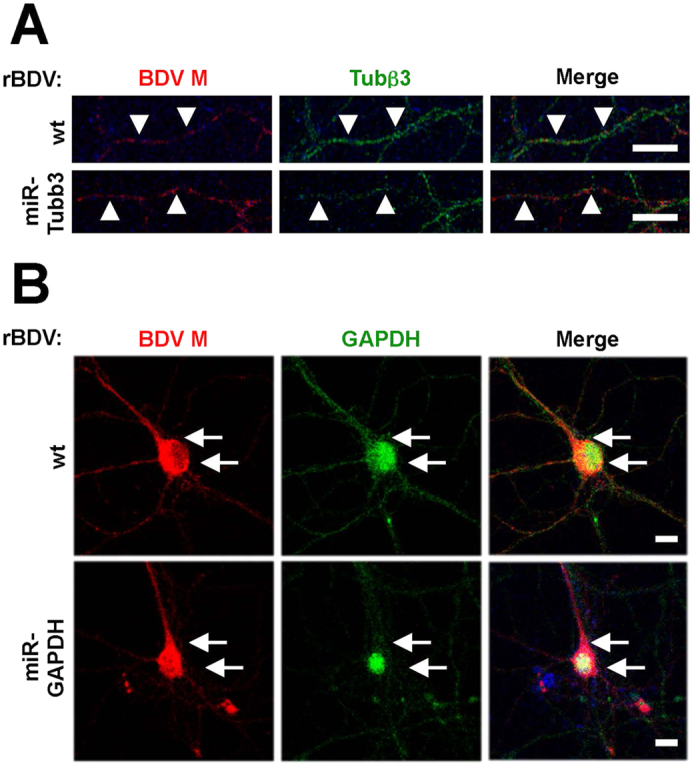
Knockdown of GAPDH and tubulin β3 expression in primary neurons. Reduction of tubulin β3 (**A**) and GAPDH (**B**) expression in mouse hippocampal neurons infected with rBDV-miR-GAPDH and rBDV-miR-Tubb3, respectively. Mouse hippocampal neurons were infected with rBDVs on day 3 *in vitro* (DIV 3). On DIV 10, the mouse hippocampal neuron was stained with indicated antibodies. Arrowheads, the vector-induced neuritis; arrows, the vector-induced neurons. Bars, 10 μm.

To determine the effectiveness of miBDV vectors for silencing endogenous mRNAs, we finally infected mouse primary hippocampal neurons with rBDV-miR-Tubb3 and rBDV-miR-GAPDH and investigated the expression level of the targeted proteins using immunofluorescence assays (IFA). As shown in Fig. 4A, neurons infected with rBDV-miR-Tubb3 appeared to reduce the level of tubulin β3 in the neurites, compared to neurons infected with wt rBDV (arrowheads). Moreover, infection with rBDV-miR-GAPDH seemed to reduce the expression of GAPDH in the somas of the neurons (Fig. 4B, arrows). These results suggest that the miBDV vector can introduce artificial miRNA against any target mRNAs of interest, effectively silencing the target genes.

## Discussion

Virus vectors provide a promising approach to delivering small RNAs *in vivo* for RNAi therapies^6^. Although retroviral and DNA viral vectors are the major systems for the delivery of RNAi molecules at present, these vectors have some risks and limitations for their application.

Recently, it has been reported that some RNA viruses, such as sindbis virus (SINV), vesicular stomatitis virus and influenza A virus (IAV), can be engineered to express miRNAs^20^,^21^,^22^,^23^. SINV is a positive-strand RNA virus and replicates in the cytoplasm. The production of SINV-derived miRNA is dependent on Drosha, which seems to be relocated to the cytoplasm following SINV infection^24^. Although this non-physiological relocation of Drosha seems to not impact on the endogenous miRNA landscape during the first 24 h post-infection, its long-term relocation has never been evaluated and might affect the landscape^24^. Furthermore, the non-canonical pathway using cytoplasmic Drosha in the processing of the SINV-derived miRNAs could affect the efficacy of miRNA processing or the function of mature miRNA, compared to that using the intranuclear cognate co-factors, such as DGCR8^24^,^25^. IAV also replicates in the nucleus and can deliver miRNAs through the canonical miRNA pathway. However, the expression of virus-derived miRNA may not be sustained because IAV infection is acute and cytopathic^20^,^23^.

In this study, we used a BDV vector to develop a novel NSV vector able to express miRNAs efficiently and persistently. BDV possesses unique features suitable for the long-term expression of miRNA molecules. Firstly, BDV replicates in the nucleus^12^,^13^,^14^,^15^, where a critical enzyme for the miRNA biogenesis is located. Secondly, BDV establishes a persistent infection in the nucleus without killing the host cells, enabling the stable expression of the transgenes for a long period of time^12^,^13^. Thirdly, the risk of integration of viral sequences into the host genome is extremely low, compared to retroviruses and DNA viruses^26^. Because of these features, BDV could be the most ideal NSV for the long-term expression of small RNAs without genomic integration of a transgene. We demonstrated that virus-derived miRNA can be expressed from a cassette in an intercistronic region of the BDV genome in primary cells and cell lines for a long time. This shows that BDV is a promising vector platform for long-term efficient expression of small RNAs.

The use of BDV vector for humans may be a concern since BDV infection has been known to induce a fatal encephalitis in some animals, including horse and sheep^27^. Although epidemiologic studies have demonstrated that BDV can infect humans, its pathogenicity to humans has remained for long unknown^28^,^29^. Recently, a new bornavirus, variegated squirrel 1 bornavirus (VSBV1), associated with fatal human encephalitis has been reported^30^. However, the phylogenetic analyses reveal that VSBV1 forms a lineage separate from that of BDV. Furthermore, the genome of VSBV1 has only ~75% identity with that of BDV, suggesting that the pathogenicity of VSBV1 cannot apply to that of BDV. On the other hand, we have previously shown that BDV P protein might be a major virulent factor^31^,^32^. Therefore, mutagenesis of virulent regions in the P protein could be a promising way to make the BDV vector safer. In addition, highly neurotropic nature of BDV might be an advantage for the use of this virus as a virus vector delivering to the central nervous system.

In the miBDV vector system, viral mRNA, which encodes the pri-miRNA sequences, could be the source of mature miRNA in the target cells, consistent with the previous studies^16^,^18^. Because the antigenomic RNA of NSV is a template for genome replication, it could be difficult to obtain the rBDV when miRNA is produced from BDV antigenomic RNA. Our successful production of rBDV encoding miRNA supports the former idea. Furthermore, the amount of BDV antigenomic RNA in cells infected with rBDV-miR-155 was comparable to that with rBDV-GFP. This may be because newly synthesized BDV antigenomic RNA molecules are co-transcriptionally packaged by the N protein into the BDV ribonucleoprotein complex (RNP)^14^, which prevents antigenomic RNA from being processed by Drosha. A previous study also demonstrates that viral genomic RNA is not sensitive to miRNA-mediated inhibition^20^.

We found that duplication of the pri-miRNA sequence in the miBDV vector system could enhance silencing activity on expression of the target gene. On the other hand, we could not detect significant differences in luciferase activity between rBDV-miR-155x2 and rBDV-155x4-infected cells, using the reporter system (Fig. 2A). We observed comparable silencing activity by transfection of miR-155 mimic or infection of the lentiviral vector expressing miR-155 (Supplemental Fig. 1C,D), suggesting that the duplication of the pri-miRNA sequence may be sufficient to achieve the threshold level of miRNA for efficient target silencing in our experimental setting. Alternatively, it is postulated that the tetrameric pri-miRNA sequences on an mRNA might destabilize the transcript or interfere with the processing of pri-miRNAs by Drosha in the nucleus. In any case, our successful production of rBDV vectors with two or four copies of the pri-miRNA sequence suggests that miBDV vectors directed against more than two targets are possible. Such miBDV vectors can inhibit the expression of functionally redundant genes in combination.

To produce artificial miRNAs of interest from the miBDV vector, we replaced both the mature miR-155 and its complementary sequences within the stem of the pri-miR-155 sequence. We used the loop region of pre-miR-155 between the stem sequences, because the loop sequences are likely to be required for efficient nuclear export of pre-miRNA^33^. We removed a few bases from the strand complementary to the miRNA of interest to create the pre-miRNA, resembling the original pre-miR-155, which might be important for its silencing efficacy (Fig. 3). Because other aspects of the miRNA precursor structure may influence its processing and/or the subsequent RNAi^34^, further optimization of the cassette sequence to express an artificial miRNA of interest is a promising way to improve this vector.

In summary, we demonstrated the potential of the miBDV vector for the long-term expression of small RNAs. The miBDV vector may provide potential solutions that overcome several concerns underlying the existing vector systems for small RNA-delivery. Further improvement of the miBDV vector, such as increasing the efficiency of miRNA processing and reducing the pathogenicity of BDV, is necessary to expand the utility of this vector system for RNAi therapies.

## Materials and Methods

### Plasmids

The BDV vector plasmid harboring an extra transcription cassette, pBDV, was generated by subcloning a cloned full-length cDNA of He/80, with the insertion of transcription initiation (S3) and termination (T2) signal sequences between the P and M genes, into pCAG-HRSV3, as described previously^10^. The miRNA precursor sequence was inserted into the *Bst*BI and *Pac*I sites of pBDV. The reporter plasmid for miRNA was generated by subcloning the firefly luciferase gene of the pGL5 plasmid (Promega), with an artificial 22-nt sequence complementary to the miRNA in the 3′UTR, into the *Eco*RI and *Xba*I sites of the pcDNA3 plasmid (Invitrogen). A pair of oligos (5′-ACC GGC TCC TAC CTG TTA GCA TTA AGT TAT TCA AGA GAT AGC TTA ATG CTA ATT GTG ATA GGG GTT TTT TGG GCC C-3′ and 5′-CGA AGG GCC CAA AAA ACC CCT ATC ACA ATT AGC ATT AAG CTA TCT CTT GAA TAA CTT AAT GCT AAC AGG TAG GAG C-3′) were annealed and inserted into the *Bbs*I sites of pRSI9-U6-(sh)-UbiC-RFP-2A-Puro (Cellecta) (pRSI9-U6-miR-155-UbiC-RFP-2A-Puro).

### Primers

The sequences of the primers used in this study are as follows:

BDV genome-specific primer, 5′-GTT GCG TTA ACA ACA AAC CAA TCA T-3′

BDV antigenome-specific primer, 5′-TGC GCT ACA ACA AAG CAA CAA CC-3′

BDV-forward primer, 5′-ATG CAT TGA CCC AAC CGG TA-3′

BDV-reverse primer, 5′-ATC ATT CGA TAG CTG CTC CCT TC-3′

miR-Tubb3-specific primer, 5′-TTA ACC TGG GAG CCC TAA TGA G-3′

miR-GAPDH-specific primer, 5′-TTG ATG ACA AGC TTC CCA TTC T-3′

let-7a-specific primer, 5′-TGA GGT AGT AGG TTG TAT AGT T-3′

### Cells

Vero cells (a monkey kidney cell line) were cultured in Dulbecco’s modified Eagle’s medium (DMEM) supplemented with 2% fetal bovine serum (FBS). OL cells (a human oligodendrocyte cell line), 293T cells (a human embryonic kidney cell line) and 293LTV cells were cultured in DMEM supplemented with 5% FBS.

### Production of rBDVs

rBDVs were produced by reverse genetics as reported previously^10^. Briefly, 293T cells were transfected with the BDV cDNA-expressing plasmids and helper plasmids expressing the BDV N, P, and L genes. The cells were passaged at 3 days post-transfection. One day later, the Vero cells were co-cultured with the transfected 293T cells and passaged every 3 days.

### Virus infection

Vero cells were infected with rBDVs at an MOI of 0.01 at 37 °C. After absorption for 1 h, the cells were washed with phosphate-buffer saline (PBS) and passaged every 3 days. Virus propagation was detected by IFA. Primary hippocampal neuron cultures were infected with rBDV at an MOI of 0.03 on DIV 3. The cultures were fixed on DIV 10.

### Production and infection of the lentiviral vector expressing miR-155

Lentiviral vector expressing miR-155 was prepared by co-transfection of pRSI9-U6-miR-155-UbiC-RFP-2A-Puro together with psPAX2 (Cellecta) and pMD2.G (Cellecta) in 293LTV cells. Vero cells were infected with the lentivirus at an MOI of 5 at 37 °C.

### Luciferase reporter assay

rBDV-infected and lentivirus-infected Vero cells were transfected with pLuc-miR-155as, pLuc-miR-Tubb3as or pLuc-miR-GAPDHas with pRL-TK (Promega). For the Dicer-knockdown experiments, rBDV-infected OL cells were transfected with siDicer (Qiagen) using Hiperfect (Qiagen). The cells were incubated for 48 h and further transfected with pLuc-miR-155as and pRL-TK. For the mouse miR-155 mimic experiments, rBDV-infected Vero cells were transfected with Syn-mmu-miR-155-5p miScript miRNA Mimic (Qiagen), pLuc-miR-155as and pRL-TK using Lipofectamine 2000 (Invitrogen). At 24 or 48 h after reporter transfection, the luciferase activity of the cells was measured using the Dual-Luciferase Reporter Assay System (Promega) according to the manufacturer’s instruction.

### Real-time RT-PCR

Total RNA was extracted from infected cells and reverse transcribed using a Verso cDNA Synthesis Kit (Thermo Scientific) with BDV genome-specific or BDV antigenome-specific primers. Quantitative real-time RT-PCR (qRT-PCR) assays were carried out using a gene-specific, double fluorescent dye-labeled probe with a Rotor-Gene Q System (Qiagen), as described previously^35^. The BDV-forward primer, BDV-reverse primer and BDV probe, 5′-FAM-AGA ACC CCT CCA TGA TCT CAG ACC CAG A-TAMRA-3′ were used in this assay. For the miR-155 detection, total RNA from the infected cells was reverse transcribed using a Taqman Reverse Transcription kit (Applied Biosystems) and the miR-155-specific RT primer (Applied Biosystems) according to the manufacturer’s instruction. Real-time RT-PCR was carried out using an Universal Master Mix (Applied Biosystems) and the miR-155-specific primers (Applied Biosystems). For the miR-Tubb3, miR-GAPDH and let-7a detection, total RNA form the infected cells was reverse transcribed using a Mir-X miRNA First-Strand Synthesis kit (Clontech) according to the manufacturer’s instruction. Real-time RT-PCR was carried out using an SYBR Advantage qPCR Premix (Clontech) and the miR-Tubb3-, miR-GAPDH- and let-7a-specific primers.

### IFA

Cells were fixed for 20 min in 4% paraformaldehyde and permeabilized by incubation in PBS containing 0.25% Triton X-100 for 10 min. After permeabilization, the cells were incubated with a mouse anti-GAPDH (Millipore), a mouse anti-tubulin β3 (Millipore), a rabbit anti-BDV N and a rabbit anti-M antibodies for 1 h. This was followed by incubation with the appropriate Alexa Fluor-conjugated secondary antibodies (Invitrogen). The cells were counterstained with 4′,6-diamidino-2-phenylindole (DAPI). A confocal laser-scanning microscope ECLIPSE Ti (Nikon Inc., Japan) was used for cell immunofluorescence imaging and data collection.

### Western blot analysis

Vero cells infected with rBDV were lysed with SDS sample buffer. The total cell lysate was subjected to SDS-PAGE and transferred onto polyvinylidene difluoride membranes (Millipore, USA). The membranes were then blocked and incubated with the primary antibodies. Antibodies used in this study were as follows: a mouse anti-BDV N (HN321), a mouse anti-GFP (Clontech) and a mouse anti-Tubulin (Sigma-Aldrich, USA) antibodies. After three washes with 0.05% Tween 20 in TBS, horseradish peroxidase-conjugated secondary antibodies (Invitrogen) were applied for 1 h at 37 °C. The bound antibodies were detected using an ECL prime Western blotting system (Amersham Pharmacia Biotech).

### Northern blot analysis

Total RNA was extracted with TRIzol reagent (Invitrogen) from Vero cells infected with rBDVs. Aliquots of 10 μg total RNA were separated on a 9% denaturing Tris-Urea gel and transferred onto nylon membranes (Roche). After UV cross-linking, the membrane was prehybridized in ULTRAhyb solution (Ambion) for 30 min at 37 °C, followed by hybridization overnight with a digoxigenin (DIG)-labeled locked nucleic acid (LNA) probe complementary to mouse miR-155 (Exiqon), miR-Tubb3 (5′-DIG-CTC ATT AGG GCT CCC AGG TTA A-3′), miR-GAPDH (5′-DIG-AGA ATG GGA AGC TTG T-3′) and human let-7a (Exiqon). The hybridized probe was detected with an alkaline phosphatase (AP)-conjugated anti-DIG antibody (Roche).

### Primary hippocampal neuron culture

Mouse hippocampal neuron cultures were prepared from embryonic day 18 C57Bl/6 mouse embryos, as previously described, with some modifications^36^. Briefly, hippocampi were dissociated by trypsinization and trituration. The neurons were plated onto poly-L-lysine-coated glass coverslips at 5,000 to 10,000 cells/cm^2^. Cultures were maintained in Neurobasal medium (Invitrogen) with 2% (vol/vol) B27 supplements (Invitrogen).

### Ethics statement

All procedures including animal studies were conducted in accordance with the guidelines for the Care and Use of Laboratory Animals of the Ministry of Education, Culture, Sports, Science and Technology, Japan. All experimental procedures were approved by the Institutional Animal Care and Use Committees (IACUC)/ethics committee of Kyoto University institutional review board (protocol number D12-11).

## Additional Information

**How to cite this article**: Honda, T. *et al.* Long-term expression of miRNA for RNA interference using a novel vector system based on a negative-strand RNA virus. *Sci. Rep.* **6**, 26154; doi: 10.1038/srep26154 (2016).

## Supplementary Material

Supplementary Information
