# Supplementary material for: Long-term expression of miRNA for RNA interference using a novel vector system based on a negative-strand RNA virus
Source: Sci Rep. 2016 May 18;6:26154. doi: 10.1038/srep26154 (PMC4870639; doi:10.1038/srep26154)
Supplement: Supplementary Information [file srep26154-s1.pdf]

Supplemental Information

Long-term expression of miRNA for RNA interference using a novel vector system based on a negative-strand RNA virus

Tomoyuki Honda[1,2*], Yusuke Yamamoto[1,2], Takuji Daito[1], Yusuke Matsumoto[1], Akiko Makino[1,3], and Keizo Tomonaga[1,2,4*]

[1]*Department of Viral Oncology, Institute for Virus Research, Kyoto University,* [2]*Department of Mammalian Regulatory Network, Graduate School of Biostudies, Kyoto University,* [3]*Center for Emerging Virus Research, Institute for Virus Research, Kyoto University,* [4]*Department of Tumor Viruses, Graduate School of Medicine, Kyoto University, Kyoto, Japan.*

*To whom correspondence should be addressed.

Department of Viral Oncology, Institute for Virus Research, Kyoto University, 53 Kawahara-cho, Shogoin, Sakyo-ku, Kyoto 606-8507, Japan

Tel: +81-75-751-4034, Fax: +81-75-751-4000

E-mail: thonda@virus.kyoto-u.ac.jp and tomonaga@virus.kyoto-u.ac.jp

Supplemental Figure legends

Supplemental Figure 1. Construction of a BDV vector plasmid containing a miRNA expression cassette. (A) Schematic representation of a BDV vector plasmid harboring the pri-miR-155 sequence in an intercistronic region between the P and M genes. (B) Schematic representation of a luciferase reporter plasmid containing a single copy of 22-nt artificial miRNA target sequences perfectly complementary to miR-155. (C) Relative luciferase activity after transfection of the luciferase reporter plasmid and mouse miR-155 mimic into Vero cells. (D) Relative luciferase activity after transfection of the luciferase reporter plasmid into Vero cells infected with the lentiviral vector expressing miR-155. Values are expressed as the mean + S.E. **, P<0.01; ****, P<0.001 (Student's t test). At least three independent experiments were performed.

Supplemental Figure 2. Dicer-mediated processing of viral pri-miR-155, possibly from viral mRNA. (A) Relative luciferase activity after transfection of the luciferase reporter plasmid into rBDV-miR-155-infected OL cells with or without siDicer treatment. (B) Quantification of BDV antigenomic RNA expression in rBDV-miR-155-infected Vero cells. The level of BDV antigenomic RNA was measured by real-time RT-PCR and standardized with the expression level of genomic RNA. Values are expressed as the mean + S.E. *, P<0.05; n.s., no significance. At least three independent experiments were performed.

Supplemental Figure 3. The sequences of synthetic pre-miRNA sequences against the GAPDH and tubulin β3 genes. (A) Arrows indicate the Drosha cleavage site. (B) Quantification of miR-Tubb3 and miR-GAPDH in rBDV-miR-Tubb3- and rBDV-miR-GAPDH-infected Vero cells. The expression of miRNAs was measured and

standardized by that of let-7. Values are expressed as the mean + S.E. ***, *P*<0.005; **** *P*<0.001. At least three independent experiments were performed.

Supplemental Figure 4. Original full-length blots before cropping. The original blots for Figures 1B (A), 1D (B), 1F (C) and 3C (D).

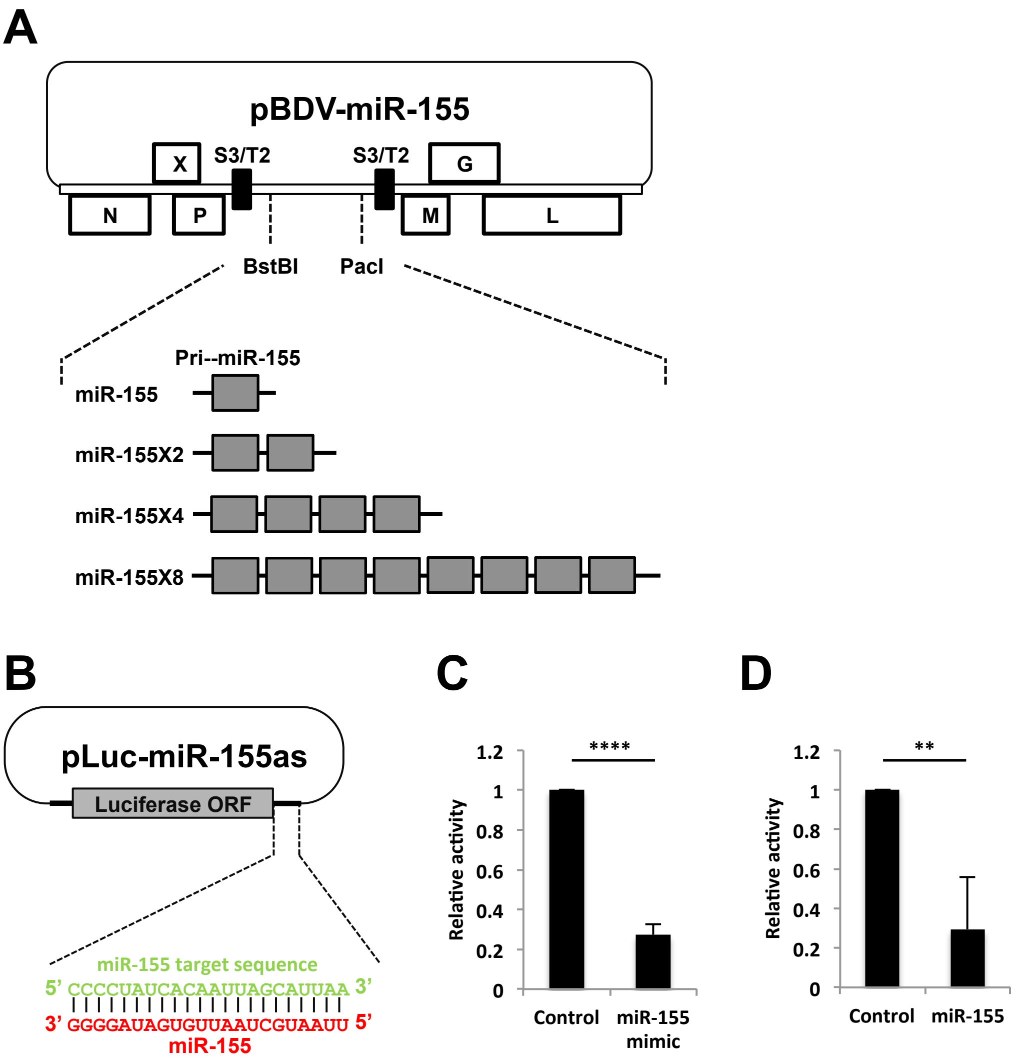

Supplemental Figure 1

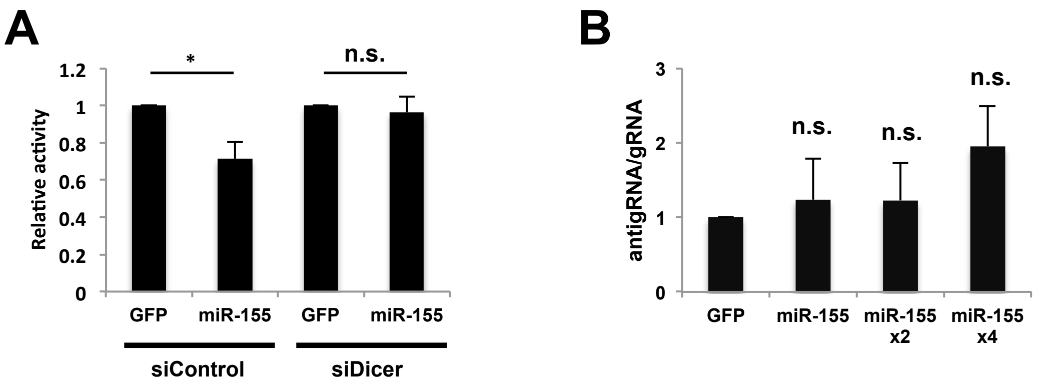

Supplemental Figure 2

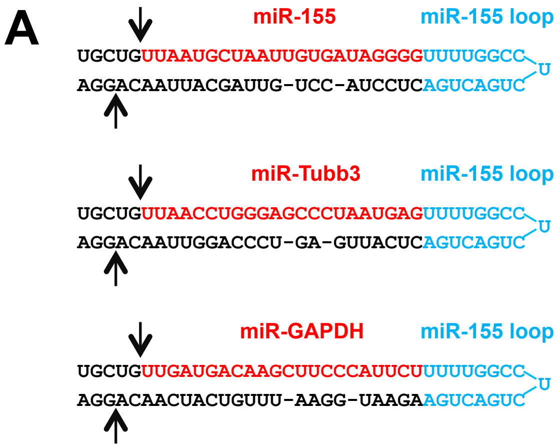

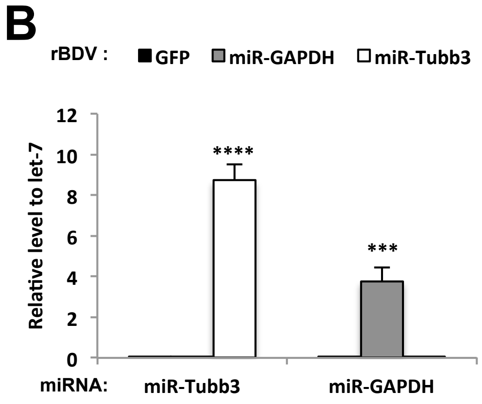

Supplemental Figure 3

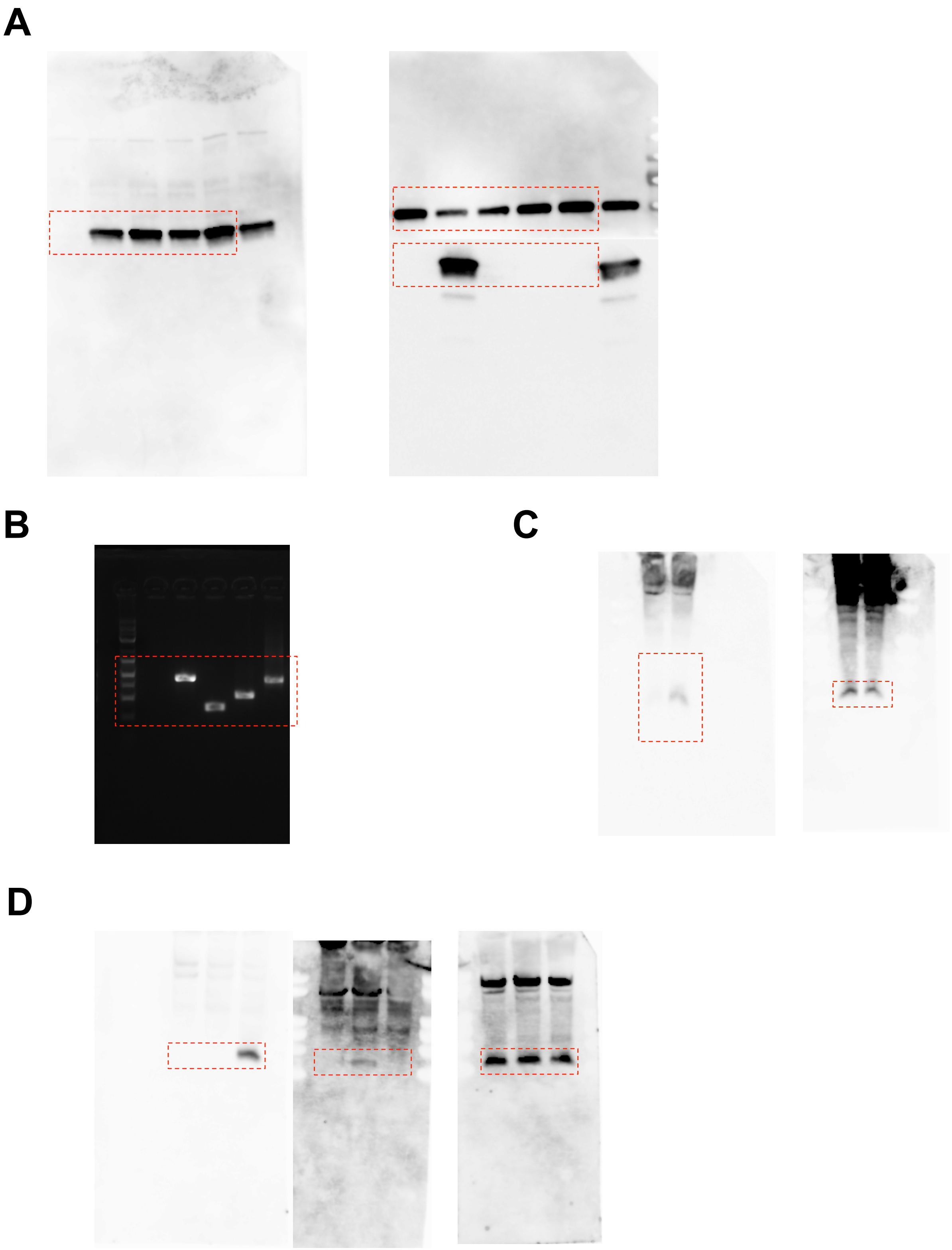

Supplemental Figure 4
